# Supplementary material for: Lived experiences of bipolar disorder and family caregiving in Pakistan
Source: Glob Ment Health (Camb). 2026 Apr 7;13:e102. doi: 10.1017/gmh.2026.10197 (PMC13202493; doi:10.1017/gmh.2026.10197)
Supplement: Umer et al. supplementary material [file S2054425126101976sup001.zip › Supplementary material_Individual with BD_Interview Guide.docx]

**Individual with BD Interview Guide: Exploring Needs for Bipolar Disorder Interventions**

Hello and thank you for coming! My name is Madeha Umer. My role on this project is student researcher. I am currently doing my PhD at University of Toronto. The purpose of this project is to gather information to improve treatment for bipolar disorder, while also making treatment more accessible. Your participation is very important to make these improvements, which will help others and can have a lasting impact on many people. The discussion will be audio-recorded. Remember, there are no right or wrong answers.

**Interview Questions**

| **Understanding of Bipolar Disorder** |
| --- |
| 1. **What is bipolar disorder? How would you describe it?** |
| بائی پولر ڈس آرڈر کیا ہے؟ آپ اسے کیسے بیان کریں گے؟ |
| 1. **What are some symptoms of bipolar disorder?**  - What do you think are other symptoms of bipolar disorder |
| بائی پولر ڈس آرڈر کی کچھ علامات کیا ہیں؟  آپ کے خیال میں بائی پولر ڈس آرڈر کی مزید کون سی علامات ہو سکتی ہیں؟ |
| 1. **What do you think is the cause of bipolar disorder?**    - - What do you think are other causes of bipolar disorder?      - Some people think bipolar disorder can be caused by a chemical imbalance, genetics, or psychological, spiritual, or social factors. What do you think? |
| آپ کے خیال میں بائی پولر ڈس آرڈر کی وجہ کیا ہے؟  آپ کے خیال میں بائی پولر ڈس آرڈر کی دیگر کیا وجوہات ہو سکتی ہیں؟  کچھ لوگوں کا خیال ہے کہ بائی پولر ڈس آرڈر کیمیائی عدم توازن، جینیات یا نفسیاتی، روحانی یا سماجی عوامل کی وجہ سے ہو سکتا ہے۔ آپ کا کیا خیال ہے؟ |
| **Understanding Experience with Bipolar Disorder** |
| 1. **Can you tell me about your experience living with bipolar disorder?**    - - How have you come to terms with your diagnosis?      - Has your illness affected your life in any significant ways (work, relationships, hobbies, etc.)? |
| کیا آپ بائی پولر ڈس آرڈر کے ساتھ اپنی زندگی کے تجربے کے بارے میں بتا سکتے ہیں؟  آپ نے اپنی تشخیص کو کیسے قبول کیا؟  کیا آپ کی بیماری نے آپ کی زندگی (کام، تعلقات، مشاغل وغیرہ) پر کسی نمایاں انداز میں اثر ڈالا ہے؟ |
| 1. **What challenges have you faced as a person living with bipolar disorder?**  - Lack of information about the illness - Stigma - Difficulty finding a treatment plan that works for you - Difficulty with self-acceptance - Financial or resource constraints (e.g., time, money, support) |
| بائی پولر ڈس آرڈر کے ساتھ زندگی گزارنے کے دوران آپ کو کیا چیلنجز کا سامنا کرنا پڑا؟  بیماری کے بارے میں معلومات کی کمی  بدنامی (اسٹیگما)  علاج کا کوئی مناسب منصوبہ تلاش کرنے میں دشواری  خود کو قبول کرنے میں دشواری  مالی یا وسائل کی کمی (مثلاً وقت، پیسہ، مدد) |
| 1. **How has bipolar disorder affected your relationships with family, friends, or colleagues?**    - - Family dynamics      - Friendships or social support      - Your own mental health and emotional well-being |
| بائی پولر ڈس آرڈر نے آپ کے خاندان، دوستوں یا ساتھیوں کے ساتھ آپ کے تعلقات کو کیسے متاثر کیا؟  خاندانی تعلقات  دوستی یا سماجی مدد  آپ کی اپنی ذہنی اور جذباتی صحت |
| **Perception of Support Needs** |
| 1. **What kind of support do you feel is currently lacking for you in managing your bipolar disorder?**    - - More information about your condition      - Knowledge about treatment options      - Guidance on how to manage day-to-day challenges      - Knowledge about preventing relapse      - Support for improving your own mental health |
| بائی پولر ڈس آرڈر کو سنبھالنے میں آپ کو کس قسم کی مدد کی کمی محسوس ہوتی ہے؟  آپ کی حالت کے بارے میں مزید معلومات  علاج کے آپشنز کے بارے میں علم  روزمرہ کے چیلنجوں کا انتظام کرنے کے لیے رہنمائی  دوبارہ علامات ظاہر ہونے کی روک تھام کے بارے میں علم  اپنی ذہنی صحت کو بہتر بنانے کے لیے مدد |
| 1. **How has your family’s involvement (or lack thereof) impacted your experience of managing bipolar disorder?**    - - Emotional support      - Practical support (e.g., helping with daily tasks, treatment adherence)      - Stress or conflict in family relationships |
| آپ کے خاندان کی شمولیت (یا اس کی کمی) نے آپ کے بائی پولر ڈس آرڈر کے انتظام کے تجربے پر کیا اثر ڈالا؟  جذباتی مدد  عملی مدد (مثلاً روزمرہ کے کاموں میں مدد، علاج کی پابندی)  خاندانی تعلقات میں دباؤ یا تنازعہ |
| 1. **What role do you think your family should play in supporting your treatment and recovery?**    - - Emotional encouragement      - Helping with medical appointments or medication      - Providing space or independence |
| آپ کے خیال میں آپ کے خاندان کو آپ کے علاج اور بحالی میں کیا کردار ادا کرنا چاہیے؟  جذباتی حوصلہ افزائی  طبی ملاقاتوں یا ادویات میں مدد کرنا  جگہ یا خود مختاری فراہم کرنا |
| "A family intervention programme for bipolar disorder aims to support both you and your family. It offers education about the disorder, teaches coping strategies, and improves communication, with the goal of creating a supportive environment for managing bipolar disorder together." |
| "بائی پولر ڈس آرڈر کے لیے فیملی انٹروینشن پروگرام کا مقصد آپ اور آپ کے خاندان دونوں کی مدد کرنا ہے۔ یہ بیماری کے بارے میں تعلیم فراہم کرتا ہے، مقابلہ کرنے کی حکمت عملی سکھاتا ہے، اور بات چیت کو بہتر بناتا ہے، تاکہ بائی پولر ڈس آرڈر کو اکٹھے سنبھالنے کے لیے ایک حمایتی ماحول پیدا کیا جا سکے۔" |
| **Perception of a Family Intervention Programme** |
| 1. **How do you think a family intervention programme could help you in managing your bipolar disorder?**    - - Could it improve your family's understanding of your needs?      - Could it help in identifying relapse signs earlier?      - Could it help in handling challenging situations within the family?      - How might it impact your own well-being? |
| آپ کے خیال میں فیملی انٹروینشن پروگرام آپ کے بائی پولر ڈس آرڈر کو سنبھالنے میں کس طرح مددگار ہو سکتا ہے؟  کیا یہ آپ کے خاندان کی آپ کی ضروریات کو سمجھنے میں مدد کر سکتا ہے؟  کیا یہ دوبارہ علامات کی نشاندہی کرنے میں جلدی مدد دے سکتا ہے؟  کیا یہ خاندان میں چیلنجنگ صورتحال سے نمٹنے میں مدد دے سکتا ہے؟  اس کا آپ کی اپنی بھلائی پر کیا اثر پڑ سکتا ہے؟ |
| 1. **How would you like a family intervention programme to be structured?**    - - Would you prefer sessions with just your family or in a group with other families?      - How often do you think these sessions should take place (weekly, fortnightly)?      - How long should the programme run (in months)?      - Who do you think should deliver this programme (e.g., a therapist, psychiatrist, social worker)? |
| آپ فیملی انٹروینشن پروگرام کی ساخت کیسے دیکھنا چاہیں گے؟  کیا آپ صرف اپنے خاندان کے ساتھ سیشن کو ترجیح دیں گے یا دوسرے خاندانوں کے ساتھ گروپ سیشن؟  آپ کے خیال میں یہ سیشن کتنی بار ہونے چاہئیں (ہفتہ وار، پندرہ روزہ)؟  پروگرام کتنی مدت کے لیے ہونا چاہیے (مہینوں میں)؟  آپ کے خیال میں اس پروگرام کو کون فراہم کرے (مثلاً تھراپسٹ، ماہر نفسیات، سماجی کارکن)؟ |
| 1. **Do you have any concerns about participating in a family intervention programme?**    - - Lack of interest from family members      - Stigma around mental health      - Your family not understanding the importance of this programme      - Financial or travel-related difficulties      - Other caregiving responsibilities within the family |
| کیا آپ کو فیملی انٹروینشن پروگرام میں حصہ لینے کے بارے میں کوئی خدشات ہیں؟  خاندان کے افراد کی دلچسپی کی کمی  ذہنی صحت کے بارے میں بدنامی  آپ کے خاندان کی اس پروگرام کی اہمیت کو نہ سمجھنا  مالی یا سفر سے متعلق مشکلات  خاندان کے اندر دیگر دیکھ بھال کی ذمہ داریاں |
| 1. **Are there any specific topics or areas that you would like a family intervention programme to address?**    - - More information about bipolar disorder      - Information about treatments and side effects of medications      - How to prevent relapse      - Ways to improve your own mental health |
| کیا کوئی خاص موضوعات یا علاقے ہیں جن پر آپ فیملی انٹروینشن پروگرام توجہ دینا چاہیں گے؟  بائی پولر ڈس آرڈر کے بارے میں مزید معلومات  علاج اور ادویات کے ضمنی اثرات کے بارے میں معلومات  دوبارہ علامات کی روک تھام کا طریقہ  اپنی ذہنی صحت کو بہتر بنانے کے طریقے |
| 1. **How comfortable do you feel with your family being involved in your treatment or understanding your condition?**    - - Sharing personal experiences      - Attending therapy or educational sessions with you      - Understanding your triggers and warning signs |
| آپ اپنے خاندان کے آپ کے علاج میں شمولیت یا آپ کی حالت کو سمجھنے کے بارے میں کتنا آرام دہ محسوس کرتے ہیں؟  ذاتی تجربات کا اشتراک کرنا  آپ کے ساتھ تھراپی یا تعلیمی سیشن میں شرکت کرنا  آپ کے ٹریگرز اور خطرے کے اشارے کو سمجھنا |
| 1. **How do you think a family intervention could help your family better understand and support you?**    - - Reducing misunderstandings or conflicts      - Promoting a more supportive environment      - Helping family members recognize early signs of relapse |
| آپ کے خیال میں فیملی انٹروینشن آپ کے خاندان کو آپ کو بہتر طور پر سمجھنے اور مدد کرنے میں کس طرح مددگار ثابت ہو سکتی ہے؟  غلط فہمیاں یا تنازعات کو کم کرنا  زیادہ مددگار ماحول کو فروغ دینا  خاندان کے افراد کو دوبارہ علامات کے ابتدائی اشارے کی پہچان کرنے میں مدد دینا |
| 1. **What types of skills or coping strategies do you think would help your family in supporting you?**    - - Communication strategies      - Emotional regulation and stress management      - Problem-solving approaches during challenging situations |
| آپ کے خیال میں آپ کے خاندان کو آپ کی مدد کرنے میں کون سی مہارتیں یا مقابلہ کرنے کی حکمت عملی مددگار ثابت ہوں گی؟  بات چیت کی حکمت عملی  جذباتی انتظام اور دباؤ کا انتظام  چیلنجنگ حالات میں مسئلہ حل کرنے کے طریقے |
| 1. **What challenges do you think might prevent your family from participating in an intervention programme?**    - - Stigma or embarrassment about mental health      - Lack of time or resources      - Conflicts within the family      - Cultural or religious beliefs around mental illness |
| آپ کے خیال میں کون سی چیلنجز آپ کے خاندان کو مداخلت کے پروگرام میں حصہ لینے سے روک سکتی ہیں؟  ذہنی صحت کے بارے میں بدنامی یا شرمندگی  وقت یا وسائل کی کمی  خاندان میں تنازعات  ذہنی بیماری کے بارے میں ثقافتی یا مذہبی عقائد |
| 1. **How could we make it easier for your family to participate in a programme?**    - - Flexible scheduling      - Remote sessions (online or phone)      - Addressing stigma or educating about mental health in culturally sensitive ways |
| ہم آپ کے خاندان کے لیے پروگرام میں شرکت کرنا کس طرح آسان بنا سکتے ہیں؟  لچکدار شیڈولنگ  دور دراز سیشن (آن لائن یا فون)  بدنامی کو حل کرنا یا ذہنی صحت کے بارے میں ثقافتی طور پر حساس طریقوں سے تعلیم دینا |
| 1. **How do you think your own mental health and well-being might change if your family was more informed and supportive through an intervention program?**    - - Less stress or conflict at home      - Feeling more understood or supported      - Greater confidence in managing your condition |
| آپ کے خیال میں اگر آپ کا خاندان ایک مداخلت پروگرام کے ذریعے زیادہ معلوماتی اور مددگار ہو تو آپ کی اپنی ذہنی صحت اور بھلائی میں کیا تبدیلی آ سکتی ہے؟  گھر میں کم دباؤ یا تنازعہ  زیادہ سمجھا ہوا یا مددگار محسوس کرنا  اپنی حالت کا انتظام کرنے میں زیادہ اعتماد |
| **Final Thoughts** |
| 1. **Do you have any other comments or suggestions regarding a family intervention program for bipolar disorder?** |
| کیا آپ کے پاس بائی پولر ڈس آرڈر کے لیے فیملی انٹروینشن پروگرام کے بارے میں کوئی دیگر تبصرے یا تجاویز ہیں؟ |
