# Supplementary material for: Lived experiences of bipolar disorder and family caregiving in Pakistan
Source: Glob Ment Health (Camb). 2026 Apr 7;13:e102. doi: 10.1017/gmh.2026.10197 (PMC13202493; doi:10.1017/gmh.2026.10197)
Supplement: Umer et al. supplementary material [file S2054425126101976sup001.zip › Supplementary material_Interview Guide_Caregivers.docx]

**Caregiver Interview Guide: Exploring Family Needs for Bipolar Disorder Interventions**

Hello and thank you for coming! My name is Madeha Umer. My role on this project is student researcher. I am currently doing my PhD at University of Toronto. The purpose of this project is to gather information to improve treatment for bipolar disorder, while also making treatment more accessible. Your participation is very important to make these improvements, which will help others and can have a lasting impact on many people. The discussion will be audio-recorded. Remember, there are no right or wrong answers.

**Interview Questions**

| **Caregiver Experience** |
| --- |
| 1. **Can you tell me about your experience as a caregiver for someone with bipolar disorder?**    - - When did you first become aware of their condition?      - How has your role as a caregiver impacted your own life (work, personal relationships, health)? |
| **آپ بائی پولر ڈس آرڈر میں مبتلا کسی کی دیکھ بھال کرنے کا تجربہ بیان کر سکتے ہیں؟**  **اشارے**  آپ کو پہلی بار کب ان کی حالت کا علم ہوا؟  بطور دیکھ بھال کرنے والے آپ کی زندگی (کام، ذاتی تعلقات، صحت) پر کیا اثرات پڑے ہیں؟ |
| 1. **What has been the most challenging part of caring for a loved one with bipolar disorder?**    - - Managing mood changes      - Lack of resources or information      - Emotional stress or burnout      - Managing your own mental health |
| **بائی پولر ڈس آرڈر میں مبتلا کسی عزیز کی دیکھ بھال میں سب سے مشکل حصہ کیا رہا ہے؟**  مزاج کی تبدیلیوں کو سنبھالنا  وسائل یا معلومات کی کمی  جذباتی دباؤ یا تھکاوٹ  اپنی ذہنی صحت کا خیال رکھنا |
| **Understanding of Bipolar Disorder** |
| 1. **How informed do you feel about bipolar disorder and its treatment?**    - - Do you feel you need more information about the condition or treatment options?      - What would you like to learn more about (e.g., medications, relapse prevention, support services)? |
| **بائی پولر ڈس آرڈر اور اس کے علاج کے بارے میں آپ کتنا باخبر محسوس کرتے ہیں؟**  کیا آپ کو اس حالت یا علاج کے آپشنز کے بارے میں مزید معلومات کی ضرورت محسوس ہوتی ہے؟  آپ مزید کس بارے میں جاننا چاہیں گے (مثلاً دوائیں، دوبارہ علامات ظاہر ہونے کی روک تھام، سپورٹ سروسز)؟ |
| 1. **How confident do you feel in recognizing signs of relapse in your loved one?**    - - Do you know what signs to look for?      - Have you ever been caught off guard by a relapse or mood episode? |
| **کیا آپ اپنے عزیز میں دوبارہ علامات ظاہر ہونے کی نشانیوں کو پہچاننے میں پراعتماد محسوس کرتے ہیں؟**  کیا آپ جانتے ہیں کہ کون سی نشانیاں دیکھنی ہیں؟  کیا آپ کو کبھی اچانک دوبارہ علامات یا مزاج کے واقعے کا سامنا کرنا پڑا؟ |
| **Impact on Family Dynamics** |
| 1. **How has your loved one’s bipolar disorder affected your family’s daily life and relationships?**    - - Has it created tension or conflict within the family?      - How do other family members respond to your loved one’s condition? |
| **بائی پولر ڈس آرڈر نے آپ کے خاندان کی روزمرہ کی زندگی اور تعلقات کو کیسے متاثر کیا؟**  کیا اس سے خاندان میں تناؤ یا تنازعہ پیدا ہوا؟  آپ کے خاندان کے دیگر افراد اس حالت پر کیسے ردعمل ظاہر کرتے ہیں؟ |
| 1. **How do you manage family responsibilities while caring for your loved one?**    - - How do you balance caregiving with work, other relationships, or personal time?      - Do you receive help from other family members? |
| **دیکھ بھال کے ساتھ خاندان کی ذمہ داریوں کو آپ کیسے منظم کرتے ہیں؟**  آپ دیکھ بھال، کام، دیگر تعلقات یا ذاتی وقت کو کیسے متوازن کرتے ہیں؟  کیا آپ کو خاندان کے دیگر افراد سے مدد ملتی ہے؟ |
| **Cultural and Social Context** |
| 1. **How has your religious and cultural background influenced your approach to caregiving?**    - - Are there cultural or religious beliefs that affect how you view mental illness?      - How do societal views on mental illness in your community affect your experience? |
| **آپ کے ثقافتی پس منظر نے آپ کی دیکھ بھال کے طریقے کو کس طرح متاثر کیا ہے؟**  کیا ثقافتی یا مذہبی عقائد ہیں جو ذہنی بیماری کے بارے میں آپ کے نظریے کو متاثر کرتے ہیں؟  آپ کی کمیونٹی میں ذہنی بیماری کے بارے میں سماجی نظریات آپ کے تجربے کو کیسے متاثر کرتے ہیں؟ |
| **Support Systems** |
| 1. **What kind of support do you receive from professionals (therapists, doctors) in managing your loved one’s condition?**  - Do you feel adequately supported by healthcare professionals? - What additional professional support would be helpful to you? |
| **آپ کو پیشہ ورانہ ماہرین (تھراپسٹ، ڈاکٹر) سے اپنے عزیز کی حالت کو سنبھالنے میں کس قسم کی مدد ملتی ہے؟**  کیا آپ صحت کی دیکھ بھال کے پیشہ ور افراد کی طرف سے مناسب مدد محسوس کرتے ہیں؟  کون سی اضافی پیشہ ورانہ مدد آپ کے لیے مفید ہو سکتی ہے؟ |
| 1. **Do you receive any emotional or practical support from friends, extended family, or community groups?**  - How do friends and family respond to your caregiving role? - Do you feel comfortable sharing your experiences with others? |
| **کیا آپ کو دوستوں، بڑھے خاندان یا کمیونٹی گروپوں سے جذباتی یا عملی مدد ملتی ہے؟**  دوست اور خاندان کے افراد آپ کی دیکھ بھال کی ذمہ داری کو کیسے دیکھتے ہیں؟  کیا آپ دوسروں کے ساتھ اپنے تجربات بانٹنے میں آرام دہ محسوس کرتے ہیں؟ |
| ***Perception of a Family Intervention Programme***  *“A family intervention programme for bipolar disorder aim to support not just the individual with the condition but the entire family. It provides education about the disorder, teaches coping strategies, and improves communication among family members. The goal is to create a supportive environment that helps everyone involved manage the challenges of bipolar disorder more effectively, leading to better outcomes for both the person with the condition and their loved ones.”* |
| بائی پولر ڈس آرڈر کے لیے فیملی انٹروینشن پروگرام کا مقصد صرف اس فرد کی مدد کرنا نہیں ہے جو اس حالت میں مبتلا ہے، بلکہ پورے خاندان کو بھی سپورٹ فراہم کرنا ہے۔ یہ پروگرام بیماری کے بارے میں تعلیم فراہم کرتا ہے، مقابلہ کرنے کی حکمت عملی سکھاتا ہے، اور خاندانی افراد کے درمیان بات چیت کو بہتر بناتا ہے۔ اس کا مقصد ایک حمایتی ماحول پیدا کرنا ہے جو بائی پولر ڈس آرڈر کے چیلنجوں سے نمٹنے میں شامل تمام لوگوں کی مدد کرے، جس سے اس فرد اور اس کے پیاروں دونوں کے لیے بہتر نتائج سامنے آئیں۔ |
| 1. **How do you think a family intervention programme could help in managing your loved one’s bipolar disorder?**    - Improved family communication and understanding    - Helping family members identify signs of relapse    - Offering coping strategies for managing difficult situations |
| **پ کے خیال میں فیملی انٹروینشن پروگرام آپ کے عزیز کے بائی پولر ڈس آرڈر کو کیسے سنبھالنے میں مددگار ہو سکتا ہے؟**  خاندان کے درمیان بہتر رابطہ اور سمجھ بوجھ  خاندان کے افراد کو دوبارہ علامات کی نشاندہی کرنے میں مدد دینا  مشکل حالات سے نمٹنے کی حکمت عملی فراہم کرنا |
| 1. **Do you think a family intervention could improve your own mental and emotional well-being? If so, how?**    - - Reducing stress or feelings of isolation      - Gaining new strategies for self-care and stress management      - Improving your family’s support in sharing caregiving responsibilities |
| **کیا آپ کو لگتا ہے کہ فیملی انٹروینشن پروگرام آپ کی ذہنی اور جذباتی فلاح و بہبود کو بہتر بنا سکتا ہے؟ اگر ہاں، تو کیسے؟**  تناؤ یا تنہائی کے احساسات کو کم کرنا  اپنی دیکھ بھال اور تناؤ کے انتظام کے لیے نئی حکمت عملیاں حاصل کرنا دیکھ بھال کی ذمہ داریوں میں خاندان کی سپورٹ کو بہتر بنانا" |
| 1. **What topics or skills do you think would be most beneficial in a family intervention programme?**    - Education about bipolar disorder and treatment options    - Learning effective communication and problem-solving skills    - Stress management or self-care for caregivers |
| **آپ کے خیال میں فیملی انٹروینشن پروگرام میں کون سے موضوعات یا مہارتیں سب سے زیادہ فائدہ مند ہوں گی؟**  **اشارے:**  بائی پولر ڈس آرڈر اور علاج کے آپشنز کے بارے میں تعلیم  مؤثر رابطے اور مسئلہ حل کرنے کی مہارت سیکھنا  دیکھ بھال کرنے والوں کے لیے تناؤ کے انتظام یا خود کی دیکھ بھال |
| 1. **How would you like a family intervention programme to be structured?**    - - Would you prefer individual family sessions or group sessions with other families?      - How often should sessions be held (weekly, fortnightly)?      - What length of programme would work best (e.g., 3 months, 6 months)? |
| **آپ فیملی انٹروینشن پروگرام کو کس طرح منظم کرنا چاہیں گے؟**  کیا آپ انفرادی فیملی سیشنز کو ترجیح دیں گے یا دیگر خاندانوں کے ساتھ گروپ سیشنز؟  سیشن کتنی بار منعقد ہونے چاہئیں (ہفتہ وار، پندرہ روزہ)؟  پروگرام کی کتنی مدت بہترین ہوگی (مثلاً، 3 ماہ، 6 ماہ)؟ |
| 1. **What concerns do you have about participating in a family intervention programme?**  - Lack of interest (family, patient) - Stigma - Family being unable to understand the importance of this programme - Financial constraints - Travel related difficulties - Caregiving responsibilities |
| **فیملی انٹروینشن پروگرام میں حصہ لینے کے بارے میں آپ کو کیا خدشات ہیں؟**  دلچسپی کی کمی (خاندان، مریض)  بدنامی (اسٹیگما)  خاندان کی طرف سے اس پروگرام کی اہمیت کو نہ سمجھ پانا  مالی رکاوٹیں  سفر سے متعلق مشکلات  دیکھ بھال کی ذمہ داریاں |
| 1. **What would make it easier for your family to participate in such a programme?**    - - Flexibility in scheduling (evenings, weekends)      - Accessibility (location, online sessions)      - Childcare, caregiving responsibilities, or travel support |
| **آپ کے خاندان کے لیے ایسے پروگرام میں حصہ لینا کیسے آسان ہو سکتا ہے؟**  شیڈول میں لچک (شام، ویک اینڈ)  رسائی (مقام، آن لائن سیشنز)  بچوں کی دیکھ بھال، دیکھ بھال کی ذمہ داریاں، یا سفر کی مدد |
| **Final Thoughts** |
| 1. **What advice would you give to ensure that caregivers like yourself benefit from a family intervention programme?**    - - Programme structure or content suggestions      - Cultural sensitivity and understanding      - Practical support for attending sessions |
| **پ کیا مشورہ دیں گے تاکہ دیکھ بھال کرنے والے افراد جیسے کہ آپ خود فیملی انٹروینشن پروگرام سے فائدہ اٹھا سکیں؟**  پروگرام کی ساخت یا مواد کے حوالے سے تجاویز  ثقافتی حساسیت اور سمجھ بوجھ  سیشنز میں شرکت کے لیے عملی مدد |
| 1. **Is there anything else you think we should consider when developing a family intervention programme for caregivers of individuals with bipolar disorder?** |
| کیا آپ کے خیال میں ہمیں بائی پولر ڈس آرڈر میں مبتلا افراد کے دیکھ بھال کرنے والوں کے لیے فیملی انٹروینشن پروگرام تیار کرتے وقت کچھ اور بھی غور کرنا چاہیے؟ |
