# Supplementary material for: Lived experiences of bipolar disorder and family caregiving in Pakistan
Source: Glob Ment Health (Camb). 2026 Apr 7;13:e102. doi: 10.1017/gmh.2026.10197 (PMC13202493; doi:10.1017/gmh.2026.10197)
Supplement: Umer et al. supplementary material [file S2054425126101976sup001.zip › Supplementary Table Participant Characteristics.docx]

**Supplementary Table 3. Sociodemographic and clinical characteristics of individuals with bipolar disorder and family caregivers**

| ID | Age (years) | BD diagnosis | Gender | Marital status | Education | Employment | Monthly income (PKR) | Duration since diagnosis (years) | Treatment history | Religion | Living arrangement | Caregiving duration (years) | Residence | Language | Access to mental health services (travel time/hrs) | Family size |
| --- | --- | --- | --- | --- | --- | --- | --- | --- | --- | --- | --- | --- | --- | --- | --- | --- |
| Caregiver 1 | 32 | - | Male | Married | None | Muazzan | 25,000 | 15 | 5–6 doctors | Islam | Own house | 15 | Landhi | Pashto | 1.5 | 7 |
| Caregiver 2 | 26 | - | Male | Unmarried | Undergraduate | Businessman | 1,500 | 3 | 2 doctors | Islam | Own house | NR | Lahore | Urdu | NR | 5 |
| Caregiver 3 | 55 | - | Female | Married | 6^th^ grade | Unemployed | NR | 2 | 2 doctors | Islam | Own house | NR | Karachi | Urdu | 2 | 6 |
| Caregiver 4 | 20 | - | Male | Unmarried | None | Unemployed | NR | 25 | 3–4 doctors | Islam | Own house | 25 | Peshawar | Pashto | NR | 10 |
| Caregiver 5 | NR | - | Female | Married | NR | Housewife | NR | 15 | NR | Islam | Own house | 15 | Karachi | Urdu | 0.5 | 2 |
| Caregiver 6 | 35 | - | Female | Married | 10^th^ Grade | Housewife | NR | 11 | NR | Islam | Own house | 11 | Mirpurkhas | Urdu | 2.5 | 6 |
| Caregiver 7 | NR | - | Female | Married | NR | Housewife | NR | 5 | 3–4 doctors | Islam | Own house | 5 | Karachi | Urdu | 0.5 | 3 |
| Caregiver 8 | NR | - | Male | Married | 12^th^ Grade | Navy officer | NR | 2 | 3–4 doctors | Islam | Own house | 1 | Not reported | Urdu | 0.5 | 7 |
| Caregiver 9 | 37 | - | Female | Married | 12^th^ Grade | Teacher | 8,000 | 3 | NR | Islam | Own house | 3 | Lahore | Punjabi | 0.75 | 6 |
| Caregiver 10 | 40 | - | Male | Separated | 9^th^ Grade | Cable worker | 30,000 | 10 | 8–9 doctors | Islam | Own house | 10 | Gujranwala | Urdu | 1.5 | 2 |
| Caregiver 11 | 60 | - | Female | Married | 2^nd^ Grade | Unemployed | 25,000 | 10 | 3 doctors | Islam | Own house | 10 | Changa Wanga | Rajputi | 2 | 13 |
| Caregiver 12 | 35 | - | Male | Married | 10^th^ Grade | Salesman | NR | 2.5 | 5 doctors | Islam | Own separate house | 2.5 | Lahore | Punjabi | 1 | 5 |
| Patient 1 | 61 | BD-II | Male | Married | 6^th^ Grade | Unemployed | NR | 2 | 2 doctors | Islam | Own house | NA | Karachi | Urdu | 2 | 6 |
| Patient 2 | 20 | BD-I | Male | Married | 9^th^ Grade | Unemployed | NR | 15 | 5–6 doctors | Islam | Own house | NA | Landhi | Pashto | 1.5 | 7 |
| Patient 3 | 24 | BD-II | Male | Unmarried | Undergraduate | Employed | 65,000 | 3 | 2 doctors | Islam | Own house | - | Lahore | Urdu | NR | 5 |
| Patient 4 | 36 | BD-I | Male | Married | 10^th^ Grade | Unemployed | 0 | 12 | 3 doctors | Islam | Family house | - | Karachi | Urdu | 0.5 | NR |
| Patient 5 | 43 | BD-I | Male | Married | 3^rd^ Grade | Salesman | 50,000 | 25 | 3–4 doctors | Islam | Own house | - | Peshawar | Pashto | NR | 10 |
| Patient 6 | 41 | BD-II | Male | Married | 8^th^ Grade | Shopkeeper | 4,000 | 11 | NR | Islam | Family house | - | Mirpurkhas | Urdu | 2.5 | 6 |
| Patient 7 | 28 | BD-I | Male | Unmarried | NR | Unemployed | NR | 5 | 2 doctors | Islam | Family house | - | Karachi | Pashto | 0.5 | 5 |
| Patient 8 | 21 | BD-I | Female | Unmarried | 12^th^ Grade | Unemployed | 0 | 3 | 3 doctors | Islam | Own house | - | Khudiyan city | Punjabi | 1 | 7 |
| Patient 9 | 43 | BD-II | Female | Married | Undergraduate | Housewife | 0 | 5 | 1 doctor | Islam | Own house | - | Lahore | Punjabi | 1 | 4 |
| Patient 10 | 30 | BD-I | Female | Married | 12^th^ Grade | Housewife | 0 | 2.5 | 5 doctors | Islam | Own separate house | - | Lahore outskirts | Punjabi | 1.5 | 5 |
| Patient 11 | 32 | BD-II | Female | Divorced | Diploma | Housewife | 0 | 7 | 2 doctors | Islam | Own house | - | Kasoor | Punjabi | 1.5 | 6 |
| Patient 12 | 24 | BD-II | Female | Married | 12^th^ Grade | Housewife | 0 | 2 | 1 doctor | Islam | Own house | - | Murree | Punjabi | 2 | 15 |
