# Supplementary material for: Lived experiences of bipolar disorder and family caregiving in Pakistan
Source: Glob Ment Health (Camb). 2026 Apr 7;13:e102. doi: 10.1017/gmh.2026.10197 (PMC13202493; doi:10.1017/gmh.2026.10197)
Supplement: Umer et al. supplementary material [file S2054425126101976sup001.zip › Supplementary Tables Illustrative Quotes.docx]

Supplementary Table .1 Illustrative verbatim quotations (Roman Urdu and English) mapped to themes, subthemes, and codes for individuals with bipolar disorder

| **Theme** | **Subtheme** | **Code** | **Quote (Roman Urdu)** | **Quote (English)** |
| --- | --- | --- | --- | --- |
| **Understanding & Explanatory Models** | Knowledge Gaps & Diagnostic Desire | Knowledge Gap | ‘Meri knowledge mein to yeh baat nahi hai ke yeh kya cheez hai.’ | ‘As far as I know, I don’t understand what this (bipolar) thing is.’ |
|  | Knowledge Gaps & Diagnostic Desire | Communication Gap | ‘Main ne nahi kaha, na un doctors ne mujhe kaha.’ | ‘I didn’t ask, nor did the doctors tell me.’ |
|  | Causal Explanations: Biological & Cultural Attributions | Cultural Belief | ‘Beemari nahi kahenge is ko; nazar aisi cheez hai jo insaan ko jeetay jee maar deti hai… agar lagti hai to insaan ko nichor deti hai.’ | ‘I wouldn’t call it a disease, ‘nazar’ (evil eye) is something that can kill someone while alive… if it strikes, it squeezes the life out of you.’ |
|  | Causal Explanations: Biological & Cultural Attributions | Medical Model Acceptance | ‘Doctors ki… chemicals jo hain woh up and down ho jate hain, jis ki wajah se… issue… aa jata hai.’ | ‘According to doctors, the chemicals go up and down, and that causes this issue.’ |
|  | Causal Explanations: Biological & Cultural Attributions | Trauma (in-law abuse & family conflict) | ‘Mujhe yeh bimaari divorce ke baad hui… susar ne… koshish ki… waalida mujhe jhooti kehti thin… main binaar ho gayi…’ | ‘I developed this illness after divorce… my father-in-law tried something… my mother-in-law called me a liar… after all this, I became childless.’ |
| **Symptom Experience & Impact** | Core Symptom Phenomenology | Treatment Adherence & Relapse | ‘Main ne doctor sahib ko yeh bataya tha ke pehlay mujhe 48 ghantay neend nahi aati thi… phir wahan se main ne dawaiyan khani chhor di… surgeon sahib ne mujhe danta… ab dobara se yeh marz shuru hua hai…’ | ‘I couldn’t sleep for 48 hours… medication helped… I stopped the pills when I went far away… the surgeon scolded me… now the illness has returned.’ |
|  | Behavioral Changes & Everyday Life Disruptions | Behavioral Consequence | ‘Jo aadmi 24 ghantay mein neend nahi karega… chirchirapan aur dimagi tawazun kamzor hoga, to har aadmi se jhagra karega.’ | ‘If someone doesn’t sleep for 24 hours… they become irritable and mentally unbalanced, so they fight with everyone.’ |
|  | Core Symptom Phenomenology | Sleep–Wake Disruption | ‘Jee, abhi bhi dekh lein na: sara din chala hoon aur saari raat nahi soya hoon… phir dopahar ko uthoon ga 12 bajay tak.’ | ‘I stay awake all day and don’t sleep at night… then I wake up in the afternoon around 12.’ |
|  | Core Symptom Phenomenology | Cognitive Disturbance | ‘Meri memory peechay chali gayi 12 saal tak… aur yeh 4–5 zubanein jo bolnay laga, iska bhi nahi pata chal raha mujhe.’ | ‘My memory has gone back about 12 years… I’ve started speaking 4–5 languages and I don’t know why.’ |
|  | Behavioral Changes & Everyday Life Disruptions | Life Impact | ‘Mushkilat bohat aayin… har kisi se jhagarna shuru kar deta hoon bila wajah… meri zindagi par bohat asar para hai.’ | ‘I start arguing with everyone for no reason… it has greatly affected my life.’ |
| **Treatment Needs and Preferences** | Care Modality and Format Preferences | Diagnostic Desire | ‘Main to yeh chahta hoon ke mera woh CT-scan… bhool jata hoon kabhi kabhi.’ | ‘What I want is to have that CT scan done… because I sometimes forget things.’ |
|  | Navigating Treatment Access & Challenges | Provider Trust | ‘Matlab yeh hai ke agar doctor acha hoga to kar lega, nahi to time-pass karne wala hoga… ja ke parchiyon par likh dega.’ | ‘If the doctor is good he’ll treat you; otherwise he’s just passing time—writing prescriptions on scraps.’ |
|  | Adherence & Relapse Prevention | Treatment Discontinuation & Relapse | ‘Jee, nafsiyati… Abbasi Hospital mein… dawaiyan hi chhor di, to zahir si baat hai dimaag wapas kharab ho gaya.’ | ‘I stopped taking the medicines… and naturally my mind became unwell again.’ |
|  | Care Modality and Format Preferences | Family Psychoeducation as Necessity | ‘Aur agar family ko bhi saath bithaya jaye… to us ka faida hai, warna patient akela reh jata hai.’ | ‘If the family is included… it helps; otherwise the patient is left alone.’ |
|  | Navigating Treatment Access & Challenges | Financial Barriers to Treatment / Health System Navigation | ‘Private hospitals… fee bohat high… medicines 2000, 3000 rupay haftay ka… ab… Guddu Chowk se sasti dawa mil jati hai… 15 din…’ | ‘Private fees and medicines are too expensive… now I go to Guddu Chowk for cheaper medicine… only 15 days’ supply, so I must go every 15 days.’ |
| **Social Dynamics & Support** | Family & Close Support Dynamics | Family Conflict | ‘Kuch gharelu tension pareshani… bachon ke saath aur biwi ke saath mujh se chugli karke biwi ko pitwaya… tum ne mere saath aur biwi bachon ke saath acha nahi kiya.’ | ‘Some domestic tensions… gossiped against me, got my wife beaten… ‘You didn’t treat me or my wife and children well.’’ |
|  | Family & Close Support Dynamics | Social Support | ‘Koi shikayat nahi hui, sab bohat achay thay… meri biwi saath deti hai, mera beta saath deta hai.’ | ‘No complaints… my wife supports me, and so does my son.’ |
|  | Experience of Social Isolation & Anticipated Stigma | Social Isolation | ‘Sab ne behnon bhaiyon ne mujh se rabta hi khatam kar diya, kehte tum pagal ho gaye ho.’ | ‘All my sisters and brothers cut off contact with me, saying ‘you’ve gone mad.’’ |
|  | Cultural Roles, Duties, and Pressures | Family Reputation Impact | ‘Ab meri betiyon ke rishte aa rahe hain, main un se sahi tareeqay se baat nahi kar sakta, khatakta hoon… Bangali boli aur Seraiki boli, yeh kaafi mix ho jati hain saari.’ | ‘Proposals are coming for my daughters, but I can’t speak properly… I mix Bengali and Seraiki dialects.’ |
|  | Cultural Roles, Duties, and Pressures | Role & Responsibility | ‘Zahir si baat hai bohat zimmedariyan hain mujh par: 3 jawan betiyan hain aur 2 betay bhi.’ | ‘I have many responsibilities: three adult daughters and two sons.’ |
| **Coping Strategies and Meaning-Making** | Coping Strategies and Meaning-Making | Religious Coping | ‘Phir yeh hua ke main ne dua karni shuru ki. Main ne Allah Pak se kaha ke Allah aap hi hain. Phir main ne tahajjud parhna shuru kiya, namaz waghera parhni shuru ki. Aahista aahista main Surah Rehman sunti thi, to us ke saath mujhe sukoon aaya. Main raat ko rozana Surah Rehman sun kar soti thi.’ | ‘Then I started praying… began listening to Surah Rehman every night to sleep, and that brought me peace.’ |
|  | Coping Strategies and Meaning-Making | Benefit from therapy | ‘Zyada yeh problem aaya tha… main ne therapies waghera lena shuru ki to us se mujhe kaafi help mili… ab depression phase to almost, Alhamdulillah, itna aata nahi hai.’ | ‘I started therapies and got a lot of help… now the depression phase almost never comes.’ |
|  | Coping Strategies and Meaning-Making | Active Help-Seeking/Information Seeking | ‘Main ne YouTube par aur Google par… beemari ke baare mein jaan’na shuru kiya… phir recovery… ke kaise recover kiya ja sakta hai?’ | ‘I started searching on YouTube and Google about this illness… and how to recover from it.’ |

Supplementary Table 2. Illustrative verbatim quotations (Roman Urdu and English) mapped to themes, subthemes, and codes for caregivers

| **Theme** | **Subtheme** | **Code** | **Quote (Urdu)** | **Illustrative quote (English)** |
| --- | --- | --- | --- | --- |
| **Lived Experience of Illness & Recovery** | Cultural & Religious Interpretations of Illness | Spiritual Healing | ‘Roohani ilaj karwaya to us se, mashAllah, behtari aa gayi. Phir samajh aaya ke aadha roohani ilaj hai aur aadha doctori ilaj.’ | ‘We tried spiritual healing and, by God’s grace, there was improvement. We then understood that care is partly spiritual and partly medical.’ |
|  | Cultural & Religious Interpretations of Illness | Faith-Based Self-Regulation | ‘Quran ki aayatein parh leta hoon to jo ghussa hota hai woh khatam ho jata hai; main apne aap ko control kar leta hoon.’ | ‘When I recite Qur’anic verses, my anger settles and I can control myself.’ |
|  | Cultural & Religious Interpretations of Illness | Religious Coping | ‘Hum log as a Christian hain, to shuru mein hum ne prayers karwayi thin.’ | ‘We are Christian, so in the beginning we arranged prayers for him.’ |
|  | Recognition and Onset of Symptoms | Hallucinations | ‘Woh kehti thi ke usay smell aana shuru ho jati thi, khushboo aana shuru ho jati thi; woh yeh mujh se share karti thi.’ | ‘She started sensing smells and would share these experiences with me.’ |
|  | Recognition and Onset of Symptoms | Self-Harm / Behavioral Disturbance | ‘Gussa aata tha to control se bahar ho jata tha; almari ka sheesha tor deta tha, aur kabhi apne aap ko bhi nuqsan pohanchata tha.’ | ‘When anger rose, he would lose control—breaking things and sometimes harming himself.’ |
| **The Multidimensional Burden of Caregiving** | Disrupted Rhythms of Daily Life | Medication Adherence | ‘Tablet pabandi se khanay laga to mazeed behtari aa gai aur yeh wali harkatein khatam ho gain.’ | ‘When he started taking medicine regularly, there was improvement, and these behaviours ended.’ |
|  | Disrupted Rhythms of Daily Life | Substance Use as Challenge | ‘Is ka dhyan rakhne mein humein jo mushkil aati hai woh yahi hai ke hum kehte hain ke bhai nasha chhor do, nasha na karo.’ | ‘The main difficulty in taking care of him is convincing him to quit drugs.’ |
|  | Disrupted Rhythms of Daily Life | Caregiving Challenges | ‘Mushkil, bohat mushkil. Raat ko neend nahi aati thi. Jab goli khatay thay to bhook bohat lagti thi, phir bohat maslay hotay thay.’ | ‘It was very difficult. He could not sleep at night, and after taking pills he would feel very hungry, which created many problems.’ |
|  | Practical Demands and Personal Sacrifice | Sacrifice/Adaptation | ‘Walid sahib kehte thay ke mere samnay ap mere bhaiyon ko kuch na kehna, na gaali dena, na gusse se baat karna. Behtar yeh hai ke yahan se nikal kar kiraye ke makaan mein chalay jayein. Phir hum ne us ki khatir apna makaan chhor kar kiraye ke makaan mein rehna shuru kar diya.’ | ‘For his sake, we left our own home and moved to a rental place.’ |
|  | Emotional Containment and Health Strain | Caregiver Health Impact | ‘Bohat zyada masail paish aatay hain. Main khud sugar aur blood pressure ki mareez hoon, aur is ki wajah se meri sugar 400, 500, 600 tak chali jati hai. Phir main raat bhar is ke saath jaagti rehti hoon. Yeh sota nahi, raat se mobile par laga hota hai.’ | ‘So many problems arise. I have diabetes and blood pressure, and because of him my sugar goes up to 400–600. I stay awake with him all night. He does not sleep and stays on his phone.’ |
| **Community, Social Networks and Coping** | Family and Community Support | Relational Strain | ‘Woh aisi harkatein kar raha hai ke chacha ki beti ne aisi baat munh par bol di. Main yeh sochta rehta hoon ke kahin woh naraz na ho jayein. Is ka khayal karte karte darr hota hai ke us ki wajah se mera chachu mujh se naraz na ho jaye.’ | ‘While caring for him, I worry my uncle might get upset with me because of his behaviour.’ |
|  | Family and Community Support | Impact on Family Life | ‘Aik routine ho gaya hai. Thorra sa asar parta hai, lekin mushkil yeh hai ke zindagi mein tangi hai. Hum bhi thorray se zindagi se naraz hain. Woh abhi kaam nahi kar sakta. Woh pehle hi kehta hai ke yeh nuqsan hoga, yeh hoga, woh hoga.’ | ‘It has become routine, but the main difficulty is financial hardship. He cannot work and often anticipates loss before anything happens.’ |
|  | Family and Community Support | Community Awareness | ‘Abhi kal bhi woh gaya tha. Do din pehle bhi paas ki dukaan par gaya tha aur kehta tha, Typhone dawa de do. Dukaan walay jaantay hain. Unhon ne phone kiya ke yeh dawa maang raha hai.’ | ‘Recently he went to a nearby shop asking for medicine. The shopkeepers know him and called us, showing local community monitoring.’ |
|  | Stigma, Concealment, and Social Withdrawal | Stigma / Impact on Children | ‘Woh bohat tang kartay hain, main tang aa jati hoon. Main sochti hoon bas yeh sahi rahein aur mere bachay theek rahein. Meri beti sochti hai ke papa school aatay hain to koi aisa na bol de ke is ke papa aise hain. Woh papa ko kehti hai, mujhay school ke paas door se chhor kar chalay jao.’ | ‘My child worries what others will say about her father and asks him to drop her at a distance from school. Stigma is affecting the child.’ |
|  | Stigma, Concealment, and Social Withdrawal | Caregiving Alone | ‘Us ke liye main ne kisi se bhi madad nahi li.’ | ‘I have not taken any help from anyone for him.’ |
| **Health System Navigation and Access** | Barriers to Access and Participation | Caregiver Initiative/Resourcefulness | ‘Hum ne kaha ke chalo, khud search karke dekh lete hain.’ | ‘We decided to research on our own.’ |
|  | Barriers to Access and Participation | Access/Barriers to Care | ‘Government hospitals mein itna rush hota hai ke doctor sahi se guideline nahi de paatay.’ | ‘In government hospitals there is so much rush that doctors cannot give proper guidance.’ |
|  | Barriers to Access and Participation | Financial Constraint | ‘Doctor ne kaha tha is ko dakhil karwa do. Main ne kaha dawa mil jaye gi, lekin bed aur kamray ka kiraya dena paray ga. Mere paas itni gunjaish nahi.’ | ‘The doctor advised admission, but the bed and room costs were unaffordable.’ |
|  | Intervention Needs and Preferences | Information Need / Care Planning | ‘Aap mujhay koi guide batayein, koi aisa planner batayein ke main is ke liye kya karoon.’ | ‘Can you give me guidance or a plan, not just medicines, so I know what to do?’ |
|  | Navigating Medical and Spiritual Systems | Pluralistic Help-Seeking | ‘Dawayen khilatay thay, phir dam karwaya. Kabhi us se sahi ho jata tha, phir dobara dawa khilatay thay.’ | ‘We tried medicines and spiritual healing, moving between both when symptoms changed.’ |
